# Prenatal Exposure to Alcohol, Tobacco, and Coffee: Associated Congenital Complications and Adverse Birth Outcomes

**DOI:** 10.3390/ijerph18063140

**Published:** 2021-03-18

**Authors:** Sarah Soyeon Oh, Sunwha Park, Young-Ah You, Yongho Jee, AbuZar Ansari, Soo Min Kim, Gain Lee, Young Ju Kim

**Affiliations:** 1 Fetal Alcohol Syndrome Prevention Center, Ewha Institute of Convergence Medicine, Ewha Womans University, Mokdong Hospital, Seoul 07985, Korea; sarahoh@ewha.ac.kr (S.S.O.); clarrissa@hanmail.net (S.P.); yerang02@naver.com (Y.-A.Y.); jyongho@ewha.ac.kr (Y.J.); abu.kim.0313@gmail.com (A.A.); zeus_0218@naver.com (S.M.K.); loveleee0102@gmail.com (G.L.); 2 Department of Obstetrics & Gynecology, College of Medicine, Ewha Womans University, Seoul 07985, Korea; 3 Medical Research Institute, College of Medicine, Ewha Womans University Seoul Hospital, Seoul 07985, Korea; 4 Advanced Biomedical Research Institute, Ewha Womans University Seoul Hospital, Seoul 07985, Korea; 5 Graduate Program in System Health Science and Engineering, Ewha Womans University, Seoul 03760, Korea

**Keywords:** fetal programming, prenatal alcohol exposure, prenatal tobacco exposure, birth complications, maternal malnutrition, adverse birth outcomes

## Abstract

A few studies to date have examined the association between prenatal exposure to alcohol, tobacco, and coffee, and congenital complications/adverse birth outcomes among South Korean populations. Thus, this study analyzed the data of 1675 Korean women with birth experience within the last 3 years for pregnancy-related health and nutritional behaviors and relative outcomes. During their pregnancies, 11.58% of the study population consumed alcohol at least once, 1.43% drank throughout all three trimesters, 1.13% smoked, 25.43% were exposed to secondhand smoking, and 28.18% consumed 3 coffees or more every day. Prenatal alcohol exposure was associated with 11.24 times increased risk of birth defects/disabilities [Odds Ratio (OR): 11.24, 95% Confidence Interval (CI) 1.07–117.86] and 10.66 times increased risk of inherited metabolic diseases (OR: 10.66, 95% CI: 1.08–104.82). Prenatal secondhand smoke exposure (OR: 1.62, 95% CI: 1.01–2.62) and coffee consumption (OR: 1.92, 95% CI: 1.22–3.03) was associated with increased risk of low birth weight. Such results were in alignment with that of previous studies and confirmed that prenatal alcohol, tobacco, and coffee exposure can have detrimental neonatal and maternal consequences.

## 1. Introduction

Often called the “neonatal window of opportunity,” early priming refers to the critical postnatal period in a newborn’s life, whereby environmental factors and nutrition during pregnancy drive immune and tissue maturation. During this period, exposure to various substances may result in increased risk of various health problems, including increased risk of non-communicable diseases such as chronic obstructive pulmonary disease, asthma, cancer, and obesity in later life [1].

Multiple Western studies have examined how prenatal exposure to alcohol, tobacco, and caffeine may be associated with a range of birth complications. Focus has been given to prenatal alcohol exposure and Fetal Alcohol Spectrum Disorders (FASDs), which are the world’s most preventable birth defects. FASDs refer to the range of mental and/or physical disabilities that occur among individuals with prenatal alcohol exposure [2]. While symptoms range from facial defects and growth problems to central nervous system abnormalities, what is undeniable is that when a women does not consume alcohol during pregnancy, FASDs are 100% preventable [3]. According to the World Health Organization (WHO), it is believed that worldwide, 9.8% (8.9–11.1) of women consume alcohol during pregnancy, and 14.6 (9.4–23.3) out of 10,000 babies are born with the severest form of FASD, i.e., Fetal Alcohol Syndrome (FAS) [4].

In recent years, scholars have remarked that most studies estimating the prevalence of FASDs have been conducted in non-Asian countries and a few, if any, have documented the prevalence of FASDs among Asian populations [5]. For example, in a systematic literature review of 69 studies comprising 6177 FASD individuals from 17 countries, only two studies from South Korea (*n* = 1) and Israel (*n* = 1) were conducted in Asia, while 25 were conducted in the United States alone [4]. Furthermore, the study conducted in South Korea only targeted special education populations where the prevalence of FAS (42.3 per 1000) is known to be substantially higher than that of the general population [5].

Such findings are alarming as, although many Asian countries traditionally discourage women from drinking during pregnancy, increased economic empowerment has resulted in greater alcohol consumption among Asian women over recent years [6]. According to a study of people living in rural China, 42% of individuals are unaware that alcoholism is a health problem [7]. In Japan, 8.1% of Japanese women consume alcohol during pregnancy and 1 out of 10,000–20,000 babies are born with FAS [8], while in Korea, 12.0% [9] to 16.4% [10] of women may consume alcohol during pregnancy [11]. In Taiwan, 26.6% of indigenous Taiwanese pregnant women drink alcohol after recognizing that they are pregnant [12].

Prenatal tobacco exposure, has been studied more thoroughly among South Korean populations; in a sample of 1090 pregnant women’s urinary cotinine measurements from 30 obstetric clinics and prenatal care hospitals in Korea, it was found that 3.03% (95% CI 1.99–4.06) smoke during pregnancy [13]. Such exposures have been associated with increased risk of children’s respiratory morbidity [14], abnormal age at menarche [15], and atopic dermatitis in childhood [16].

Lastly, as emphasized by scholars of fetal programming, maternal nutritional status is a critical factor for the health of the developing fetus [17]. During this critical period, a fetus’s tissues and organs are created, which means that any maternal stimulus during this time can result in lifelong damages to structural and physiological functions.

Among South Korean populations, maternal coffee intake has been associated with increased risk of early pregnancy bleeding [18] and the risk of abortions [19], while abnormal (underweight, overweight, or obese) pre-pregnancy body mass index (BMI) has been associated with increased risk of preterm birth, preeclampsia, cesarean section deliveries, and gestational diabetes [19]. Thus, we hypothesize that certain maternal nutritional behaviors, including coffee consumption, dieting, and the consumption of junk foods are associated with increased risk of birth defects and/or congenital abnormalities in our study population.

Ultimately, this study aims to investigate the under-researched prevalence of alcohol consumption among pregnant women in South Korea, as well as the association between prenatal exposures to certain health-related behaviors (alcohol, coffee, junk-food, dieting, smoking, second-hand smoking) and adverse birth outcomes.

## 2. Materials and Methods

We requested data from the 2013 Korea Institute for Health and Social Affairs (KIHASA) Pregnancy Planning Survey, which surveyed a total of 1675 Korean women with childbirth experiences between 2011 and 2013. KIHASA’s aim was to gain an understanding of pregnancy behaviors among a nationally representative sample of Korean women. Convenience sampling was used to recruit women between the ages of 20 to 40 around the country. Trained surveyors were employed to conduct face-to-face interviews with the recruited women, who were required to answer questions related to their prenatal health behaviors and relative birth outcomes.

Health-related behaviors during pregnancy, as asked in the survey, were defined as any alcohol consumption during pregnancy (yes, no), any smoking during pregnancy (yes, no), and any secondhand smoking as a result of exposure from a smoking family member or colleague (yes, no). Nutritional behaviors during pregnancy were defined as any dieting during pregnancy, which resulted in the restriction of food by at least 50% (yes, no); any drinking of coffee during pregnancy, as defined by 3 coffees or more per day (yes, no); and regular junk food consumption during pregnancy, as defined by the consumption of coke, instant noodles, pizza etc. more than 4 times per week. Congenital complications were defined as any diagnosis by a medical professional of congenital birth defects/disabilities or inherited metabolic diseases (yes, no) of the child in question.

Regarding our statistical analyses, to examine the association between prenatal health-related/nutritional behaviors and congenital complications, we controlled for numerous maternal and infant-related characteristics including maternal age of delivery, household income at the time of delivery, educational attainment, pre-pregnancy BMI, past miscarriage experience, gestational diabetes/hypertension as diagnosed by a medical professional, parity, and gestational age of the infant at his/her time of birth.

Also, we examined the frequencies and percentages of each categorical variable and performed χ2 tests to examine the distribution of adverse birth complications for each outcome variable. Subsequently, we performed multivariate logistic regression to examine the association between health-related/nutritional behaviors during pregnancy and congenital complications (birth defects/disability) and inherited metabolic diseases. Statistical significance was determined by a two-tailed test with a *p*-value of 0.05 as the threshold. All statistical analyses were performed using SAS software, version 9.4 (SAS Institute, Cary, North Carolina, USA). All methods were performed in accordance with relevant guidelines and regulations.

This study was approved by the Yonsei University Health System Institutional Review Board (IRB No. Y-2020–0239), which waived the requirement for written informed consent because of the retrospective and anonymous nature of study data.

## 3. Results

Table 1 presents the general characteristics of the study population with regard to congenital complications. Our study population consisted of 1675 women with childbirths within the last 3 years, among which 1.19% resulted in birth defects/disability, 1.43% resulted in inherited metabolic disease, and 7.38% resulted in low birth weight. Regarding health/nutritional behavior, 1.43% of our study population consumed alcohol throughout their entire pregnancy, 1.13% smoked, 25.43% were exposed to secondhand smoke, 2.93% dieted, 28.18% drank two coffees or more daily, and 16.18% ate junk food regularly.

Table 2 presents the results of the logistic regression analysis examining the association between health-related/nutritional behaviors during pregnancy and congenital complications as well as adverse birth outcomes. Compared to babies with no prenatal alcohol exposure, exposed babies had 11.24 times increased risk of birth defects/disabilities (OR: 11.24, 95% 1.07–117.86) and 10.66 times increased risk of inherited metabolic disease (OR: 1.66, 95% CI: 1.08–104.82). Compared to babies with no prenatal secondhand smoke exposure, exposed babies had 1.62 times increased risk of low birth weight (OR: 1.62, 95% CI: 1.01–2.62). Compared to babies with no prenatal caffeine exposure, exposed babies had 1.92 times increased risk of having low birth weight (OR: 1.92, 95% CI: 1.22–3.03).

## 4. Discussion

Our findings show that different maternal behaviors have varying effects on congenital complications and adverse birth outcomes. To our knowledge, this is one of the first published studies to employ data from the KIHASA Pregnancy Planning Survey. It is also one of the first studies in South Korea and Asia to investigate early priming and analyze prenatal exposure to alcohol, tobacco, and/or other health-related behaviors on infant outcomes. Like previous studies, our study found a strong association between prenatal alcohol exposure and the risk of birth defects/disabilities among South Korean populations. Such studies have found that alcohol consumption during pregnancy can increase risk of birth defects by around 5-fold (AOR: 4.6, 95 CI: 1.5–14.3) [20].

In-utero alcohol exposure has consistently been associated with disrupted fetal brain development, as well as a wide range of neurobehavioral outcomes (FASDs) [21]. It was not surprising that prenatal alcohol exposure was associated with congenital anomalies, despite being adjusted for by other risk factors including older maternal age, low socioeconomic status, abnormal BMI, and gestational diabetes/hypertension. Congenital anomalies such as ventricular septal defects, cleft lips with cleft palates, low-set ears, hydrocephalies, and Down syndrome, have been associated with a number of predictors including prenatal exposure to alcohol [22], chemotherapy [23], cannabis [24], and methadone [25]. While first trimester alcohol exposure was not associated with any adverse birth outcomes in our study population, previous studies have emphasized that first trimester drinking alters placental perfusion and fetal oxygen availability, which affects fetal growth and development [26]. More studies are needed with a longitudinal, nationally representative sample to verify such results.

In our study, the consumption of three or more coffees per day was associated with increased risk of low birth weight by around twofold, which was in alignment with previous studies using the Osaka Maternal and Child Health Study (OMCHS) prospective cohort, where maternal caffeine intake was associated with a 1.28 times increased risk of preterm birth per 100 mg/d of caffeine [27]. There are frequent reports of significant dose-response associations between coffee consumption and adverse birth outcomes, implying that “moderate” caffeine consumption during pregnancy as advised by healthcare professionals may not be safe [28]. As expected, compared to babies with no prenatal secondhand smoke exposure, babies with prenatal secondhand smoke exposure had increased risk of low birth weight, as did babies born to mothers who dieted during their pregnancies. Such results were in alignment with previous studies, which have found that secondhand smoke exposure increases risk of preterm birth by more than twofold, as well as admission to a Neonatal Intensive Care Unit (NICU) by nearly sevenfold [29].

However, our study has several limitations that must be considered when interpreting results. Inherited metabolic diseases are mostly autosomal recessive conditions and their presence is determined by the laws of Mendelian inheritance rather than environmental exposures. Thus, more studies are required to identify the exact mechanisms behind the association between prenatal alcohol exposure and inherited metabolic diseases to support the results of our analyses. Similarly, the definitions of certain health/nutrition-related behaviors including “dieting” or “consuming junk food on a regular basis” were not defined to the extent that they hold clinical validity; in future studies, survey-makers should take precautions to define such behaviors clinically so that there is more validation in research that employs such data.

Public access to more recent versions of the survey is unavailable; resulting in the current dataset being from 2013. This results in various biases in interpreting our results, especially because knowledge about FASDs, and general alcohol consumption behaviors have been changing dramatically in South Korea in recent years [30]. Likewise, our study was retrospective in manner, and as is the case with all studies that require recall, may have resulted in recall loss and bias from study subjects attempting to remember their behaviors from the past. This survey was conducted in 2013, so for women who gave births up to two or three years prior to the survey date in 2011 or 2012, there would be increased risk of recall bias which could not be controlled for.

Also, the survey instrument did not ask individuals to specify the type of congenital complication in question and only asked for “any (general) diagnosis by a medical professional of congenital birth defects/disabilities, and/or inherited metabolic diseases (yes, no) of the child in question.” Specification of the exact type of congenital complication or metabolic disease in detail would enrich future studies. In our investigation, a total of 44 (2.62%) infants suffered from congenital complications and 122 (7.28%) infants had low birth weight. This is slightly lower than the prevalence of congenital abnormalities reported in previous studies (446.3 per 10,000 births (95% CI: 444.0–448.6)) [31]. It is recommended that future studies explore this manner in-depth as our study, which was self-reported and broad in terms of this outcome, is likely to be an underreport. A more gender-specific approach towards assessing infant outcomes would also increase statistical validity.

Lastly, because subjects were interviewed regarding a sensitive issue, i.e., alcohol/tobacco use during pregnancy, these statistics are likely to be underreports. In a 2010 study of 1090 South Korean women, 0.55% self-reported that they smoked but 3.03% were found to have actually smoked when urinary cotinine levels were measured (>100 ng/mL) [13]. Such behaviors are commonly “hidden” so future studies should attempt to verify study results via biochemical or medical procedures [32]. The number of individuals surveyed in our study was inadequate to give a comprehensive overview of national statistics and make certain statistical associations. In our study population, there were only 19 women who claimed to smoke during pregnancy, of which none gave birth to infants with congenital complications. Only one woman who drank throughout all three trimesters of her pregnancy gave birth to an infant with congenital complications. Although this study is valuable because it is the first of its kind among a South Korean population, more studies with greater, nationally representative population samples are required for an accurate understanding of our study question.

## 5. Conclusions

Overall, such findings reveal that exposure to alcohol, secondhand smoking, and caffeine can have adverse maternal and neonatal outcomes. As expected, prenatal alcohol exposure may increase risk of various congenital complications like birth defects or disabilities and inherited metabolic diseases, while secondhand smoking, dieting, and drinking coffee may result in increased risk of low birth weight. Our study reveals that among South Korean women, such behaviors should not be encouraged as they may have severe consequences for both the mother and expected child.

To our knowledge, this is the first study in South Korea to measure the association between maternal alcohol, tobacco, and caffeine consumption with adverse infant outcomes. Our study was limited because not much data in our country is available regarding this issue. Future researchers are advised to continue surveying and measuring the pregnancy behaviors of South Korean women, with a focus on exposure timing and biochemical verification. Clinicians and expecting parents are advised to be aware of these risks so that such behaviors can be avoided at all costs during pregnancy.

## Figures and Tables

**Table 1:** General Characteristics of study participants and health-related/nutritional behaviors during pregnancy.

	Total		Congenital Complications (*n* = 44)						Low Birth Weight (*n* = 122)		
Parameters			Birth Defect/Disability			Inherited Metabolic Disease					
	*n*	%	*n*	%	*p*-Value	*n*	%	*p*-Value	*n*	%	*p*-Value
Maternal/Infant Characteristics											
Age of delivery											
≤25	88	5.25	0	0.00	0.679	0	0.00	0.3563	4	3.28	0.0412
26–30	496	29.61	5	25.00		9	37.50		38	31.15	
31–35	794	47.4	11	55.00		13	54.17		48	39.34	
>35	297	17.73	4	20.00		2	8.33		32	26.23	
Household income											
Low	273	16.3	1	5.00	0.1908	5	20.83	0.4603	16	13.11	0.0264
Medium	1281	76.48	16	80.00		16	66.67		90	73.77	
High	121	7.22	3	15.00		3	12.50		16	13.11	
Educational attainment											
Bachelor’s degree	525	31.34	6	30.00	0.8963	9	37.50	0.5125	39	31.97	0.8774
>Bachelor’s degree	1150	68.66	14	70.00		15	62.50		83	68.03	
Pre-pregnancy BMI											
Normal (<25)	768	45.85	12	60.00	0.429	11	45.83	0.7031	70	57.38	0.0199
Overweight (25–29)	760	45.37	7	35.00		12	50.00		41	33.61	
Obese (≥30)	147	8.78	1	5.00		1	4.17		11	9.02	
Gestational diabetes	65	3.88	7	35.00		8	33.33		11	9.02	
Gestational hypertension	29	1.73	6	30.00		9	37.50		16	13.11	
Miscarriage experience	22	1.31	0	0.00		0	0.00		1	0.82	
Gestational age											
≤37 weeks (preterm)	227	13.55	5	25.00	0.3165	7	29.17	0.0778	77	63.11	<0.0001
38–42 weeks	1444	86.21	15	75.00		17	70.83		45	36.89	
>42 weeks	4	0.24	0	0.00		0	0.00		0	0.00	
Parity											
0	937	55.94	8	40.00	0.3423	9	37.50	0.1492	71	58.20	0.8669
1	598	35.7	10	50.00		13	54.17		41	33.61	
≥2	140	8.36	2	10.00		2	8.33		10	8.20	
Health/nutrition-related behavior											
Drinking alcohol *	24	1.43	1	5.00		1	4.17		1	0.82	
Smoking	19	1.13	0	0.00		0	0.00		1	0.82	
Secondhand smoking	426	25.43	4	20.00		8	33.33		42	34.43	
Dieting **	49	2.93	0	0.00		1	4.17		1	0.82	
Drinking coffee ***	472	28.18	2	10.00		7	29.17		50	40.98	
Eating junk food ****	271	16.18	2	10.00		6	25.00		14	11.48	
Total	1675	100	20	1.19		24	1.43		122	7.28	

* Throughout entire pregnancy (prevalence of first-trimester drinking only: *n* = 194; 11.58%), ** Food restriction by at least 50.0%, *** More than two coffees per day, **** Coke, instant noodles, pizza, etc. more than 4 times per week.

**Table 2:** Logistic regression analysis of association between maternal factors and congenital complications/low birth weight.

	Congenital Complications						Low Birth Weight		
Parameters	Birth Defect/Disability		*p*-Value	Inherited Metabolic Disease		*p*-Value			*p*-Value
	OR	95% CI		OR	95% CI		OR	95% CI	
Health/Nutrition-related Behavior *									
Drinking alcohol	11.24	(1.07–117.86)	<0.0001	10.66	(1.08–104.82)	0.0145	1.07	(0.13–9.14)	0.9656
Smoking	n.a.						1.25	(0.03–56.53)	0.3910
Secondhand smoking	1.04	(0.32–3.45)	0.4512	2.3	(0.82–6.45)	0.4982	1.62	(1.01–2.62)	<0.0001
Dieting	n.a.			2.16	(0.23–20.23)	0.1564	0.41	(0.05–3.42)	0.9078
Drinking coffee	0.18	(0.03–0.96)	<0.0001	0.69	(0.23–2.02)	0.1985	1.92	(1.22–3.03)	0.0543
Eating junk food	0.47	(0.09–2.59)	<0.0001	1.47	(0.47–4.54)	<0.0001	0.66	(0.34–1.29)	0.5655

* Adjusted for gestational age, maternal age, income, education level, BMI, parity, gestational diabetes, gestational hypertension, previous miscarriage experience, and other health/nutrition-related behaviors.

## Data Availability

Restrictions apply to the availability of these data. Data was obtained from the Korea Institute for Health and Social Affairs (KIHASA) and are available at https://www.kihasa.re.kr/english/main.do with the permission of KIHASA.
